# Why High-Volume Post-Dilution Hemodiafiltration Should Be the New Standard in Dialysis Care: A Comprehensive Review of Clinical Outcomes and Mechanisms

**DOI:** 10.3390/jcm14144860

**Published:** 2025-07-09

**Authors:** Stefano Stuard, Franklin W. Maddux, Bernard Canaud

**Affiliations:** 1 Global Medical Office, Fresenius Medical Care, Waltham, MA 02451, USA; frank.maddux@freseniusmedicalcare.com; 2 School of Medicine, Montpellier University, 9 Rue des Carmelites, 34090 Montpellier, France; canaudbernard@gmail.com

**Keywords:** hemodiafiltration, clinical outcomes, convection volume, anemia, inflammation, cognitive function

## Abstract

The management of end-stage kidney disease (ESKD) poses a substantial clinical and economic challenge, characterized by a growing patient burden, rising healthcare costs, and persistent unmet needs to enhance survival outcomes and quality of life. **Background/Objectives:** Conventional high-flux hemodialysis (HD) remains the dominant form of renal replacement therapy for ESKD but is still associated with substantial morbidity and mortality. High-volume post-dilution online hemodiafiltration (HVHDF) offers a promising alternative by enhancing the convective removal of uremic toxins. **Methods:** We conducted a narrative review of randomized controlled trials, meta-analyses, real-world cohort studies, and registry analyses published between 2010 and 2024. Evidence was categorized into short-term, medium-term, and long-term outcomes, including hemodynamic stability, inflammation, anemia, infection risk, cardiovascular events, cognitive decline, quality of life, and survival. **Results:** HVHDF improves short-term outcomes by enhancing toxin clearance, stabilizing blood pressure, reducing inflammation and oxidative stress, and improving anemia management. Medium-term benefits include improved nutritional status, reduced hospitalizations related to infections, and improved neurological and immune function. Long-term data from major trials (e.g., ESHOL, CONVINCE) and large real-world studies show consistent reductions in all-cause and cardiovascular mortality, particularly with convection volumes ≥ 23 L/session. A clear dose–response relationship supports the clinical relevance of convection volume targets. HVHDF has also shown benefits in preserving cognitive function and enhancing health-related quality of life. **Conclusions:** Strong and converging evidence supports HVHDF as a superior dialysis modality. Given its survival benefits, better tolerance, and broader impact on patient outcomes, HVHDF should be considered the new standard of care in dialysis, especially in light of the recent regulatory approval of the machine that provides the ability to perform HDF in the United States.

## 1. Introduction

Conventional hemodialysis (HD) remains the most commonly used renal replacement therapy (RRT) for patients with end-stage kidney disease (ESKD). Despite advances, conventional hemodialysis (HD) remains associated with persistently high morbidity and mortality. According to the United States Renal Data System 2024 Annual Data Report, the all-cause mortality rate among HD patients in 2022 was 145.6 per 1000 person-years, nearly unchanged from 2012 [1]. In response to these limitations, online post-dilution hemodiafiltration (HDF) and, more recently, high-volume post-dilution hemodiafiltration (HVHDF) have emerged as promising alternatives [2,3,4,5]. By enhancing the clearance of middle- and large-molecular-weight uremic toxins and improving treatment tolerance, HVHDF has been associated with better clinical outcomes. Over two decades of comparative research have confirmed that HDF, particularly the HVHDF with convection volumes ≥ 23 L/session, delivers meaningful clinical advantages [2,3,6,7]. Recent reviews have summarized these benefits (Table 1) [8,9,10,11,12]. Considering the dilution factor, a minimum convection volume of 46 L per session would be required for predilution HDF [13,14]: Kikuchi et al. estimated that the optimal substitution volume for improved survival in patients undergoing pre-dilution HDF was 50.6 L/session [15]. This manuscript provides a critical synthesis of the clinical evidence supporting HDF, with a specific focus on HVHDF. Outcomes are reviewed across three temporal dimensions: Short-, mid-, and long-term outcomes, to deliver a comprehensive, evidence-based perspective supporting the broader adoption of HVHDF in clinical practice. Its increasing uptake in Europe and recent regulatory approval in the United States underscore its growing clinical relevance.

## 2. Materials and Methods

This review presents a structured narrative synthesis of clinical outcomes associated with online HDF compared to conventional high-flux hemodialysis, focusing on adult patients with ESKD. It includes a critical appraisal of peer-reviewed published articles on randomized controlled trials (RCTs), meta-analyses, individual patient data (IPD) meta-analyses, registry studies, and large-scale real-world evidence (RWE).

Outcomes were stratified into three domains: Short-, mid-, and long-term outcomes. A targeted literature search was conducted using PubMed/MEDLINE (U.S. National Library of Medicine, Bethesda, MD, USA; https://pubmed.ncbi.nlm.nih.gov, accessed on 9 June 2025) and the Cochrane Library (Wiley, Hoboken, NJ, USA; https://www.cochranelibrary.com, accessed on 9 June 2025). Search terms included “hemodiafiltration,” “high-flux hemodialysis,” “convection volume,” “mortality,” “anemia,” “inflammation,” and “quality of life.”

Additional references were identified through citation tracking and expert consultation. Eligible studies included RCTs, IPD meta-analyses, registry-based observational studies, and real-world cohort analyses reporting clinical outcomes (morbidity, mortality, quality of life, or biochemical parameters).

Exclusion criteria were non-comparative case series, and studies lacking defined treatment parameters (e.g., undefined convection volumes).

## 3. Short-Term Intermediate Outcomes

HDF offers several physiological and biochemical advantages over conventional HD, particularly in terms of solute clearance efficiency.

### 3.1. Enhanced Toxin Clearance

In 2003, the European Uremic Toxins working group classified uremic toxins based into three categories based on physicochemical properties affecting dialytic removal [16,17,18]: small water-soluble compounds (WSCs, <500 Da), middle-molecular-weight substances (MMWs, 0.5–40 kDa), and protein-bound uremic toxins (PBUTs).

By combining diffusion and convection, HVHDF enhances clearance across all solute classes, notably in small solutes (e.g., urea, creatinine, and phosphate).

HVHDF enhances urea (60 Da) removal with efficacy proportional to substitution volume [19,20,21,22,23,24]. The DOPPS study showed superior Kt/V urea in patients receiving 15–24.9 L/session of substitution fluid versus those on standard HD [20].

Optimized HVHDF prescription, utilizing automated ultrafiltration and substitution control, achieves higher clearances of small- and middle-molecule clearance without increasing dialysis fluid consumption, thus improving both efficiency and environmental sustainability [25].

Phosphate (95 Da): Clearance is increased by 15–20%, potentially reducing phosphate binder use [26,27,28,29,30,31,32]. However, the impact on predialysis serum phosphate is modest (<15%), influenced by rebound kinetics and improved appetite in HDF-treated patients [10,31,33,34].

Middle and large molecule clearance: HVHDF significantly enhances the removal of MMWs such as β2-microglobulin (β2M) and tends to improve the elimination of PBUTs, which are poorly removed by HD but are associated with inflammation and cardiovascular risk.

Table 2 summarizes MMWs’ toxins and PBUTs, for which increased clearance with HDF vs. high flux has been demonstrated, along with their clinical relevance.

### 3.2. Improved Hemodynamic Stability

Efforts to enhance outcomes in chronic dialysis patients increasingly emphasize reducing intradialytic and peridialytic hemodynamic instability. Intradialytic hypotension (IDH), which was once considered benign, is now recognized as a serious clinical event. Both symptomatic and subclinical IDH episodes contribute to myocardial stunning, the hypoperfusion of vital organs (e.g., brain, gut, and kidneys), and are associated with symptomatic distress, reduced dialysis efficacy, increased vascular access thrombosis, the loss of residual kidney function, cardiovascular events, and higher mortality risks [66]. These adverse outcomes result largely from repeated organ hypoperfusion and cumulative tissue and organ injury [67,68,69].

Numerous studies, including several RCTs, show that HVHDF is associated with a lower incidence of IDH compared to conventional HD, independently of sodium balance effects [34,70,71,72,73,74]. HDF addresses multiple mechanisms contributing to hemodynamic instability [75,76]: (a) Sodium handling and the Gibbs–Donnan effect: High convective transport during HVHDF, balanced by online substitution fluid, induces a mild hypertonic gradient. Albumin binding and the Donnan effect reduce sodium concentration in the ultrafiltrate, raising plasma osmolality and enhancing plasma vascular refilling from the interstitial space. This supports effective volemia and stabilizes blood pressure during dialysis [75,77,78]. Despite large substitution volumes, sodium mass balance is well preserved [79], with no evidence of fluid overload or dysnatremia [73]. In a multicenter cohort study, Chazot et al. confirmed no increased risk of volume overload with post-dilution HVHDF [80]. However, improved sodium balance alone does not explain the hemodynamic benefits observed with HDF compared to high-flux HD [9,81]. (b) Endothelial effects of substitution fluid: The use of isotonic bicarbonate-buffered substitution fluid, with superior hemocompatibility, may stabilize endothelial function, support vascular tone, and facilitate plasma refilling, thereby contributing to improved hemodynamic stability [8,82,83]. (c) Thermal balance: Heat loss from the extracorporeal circuit causes mild core cooling, which has been linked to better blood pressure stability during dialysis [72,84]. This benefit diminishes under isothermic conditions, suggesting additional mechanisms, particularly endothelial modulation, are involved [85]. (d) Inflammatory modulation: Ultrapure dialysate and sterile substitution fluid are more effective in reducing systemic inflammation than high-flux HD [60,86,87]. While inflammation is implicated in intradialytic hypotension (IDH), the direct causal relationship remains speculative. (e) Supportive and indirect factors: HVHDF is associated with improved anemia management [88,89,90], better nutritional status [90,91], enhanced physical functioning [91,92,93,94,95], and preservation of residual kidney function [96]. These benefits may indirectly enhance cardiovascular resilience and overall hemodynamic tolerance.

Supporting evidence from recent studies. While early RCTs showed mixed results, as highlighted by the European Dialysis (EuDial) Working Group, newer studies and analyses with a better methodology suggest a protective effect of HDF on IDH [97,98]. These limitations have been addressed in a recent study using real-world data. In a target emulated study (Zoccali et al.), using real-world data from 4072 incident HD patients, the study showed HDF modifies the relationship between dialysis and IDH risk, acting as a mitigating factor, despite not being explicitly designed to assess IDH [97]. The HOLLANT study demonstrated that HVHDF significantly reduced IDH compared to conventional HD, resulting in better intradialytic blood-pressure stability [98]. Italian Convective Study (Locatelli et al.): HDF reduced symptomatic IDH by 51% (*p* < 0.001), outperforming hemofiltration and HD. Predialysis systolic blood pressure increased significantly in HDF patients, reflecting enhanced vascular stability [11,12,70].

In brief, HVHDF, improves intradialytic hemodynamic stability through multiple complementary mechanisms: osmotic-driven plasma refilling, endothelial effects, thermal balance modulation, and reduced inflammation. Recent well-controlled studies increasingly support its role in preventing IDH, systemic stress, and enhancing cardiovascular protection.

### 3.3. Reduction in Intradialytic Cramps

Intradialytic muscle cramps are a frequent and distressing complication of HD, affecting 33–86% of patients with ESKD and occurring in 5–20% of sessions [99,100,101]. These painful events arise toward the end of a session, potentially leading to premature termination, compromised dialysis adequacy, and diminished quality of life [102]. The pathophysiology is multifactorial and not fully elucidated, but key contributors include intradialytic hypotension (IDH), excessive ultrafiltration rates (UFRs), inaccurate dry weight assessment, altered plasma osmolality, electrolyte imbalances, and perfusion [103,104,105,106]. Additional contributors may include vitamin deficiencies, elevated serum leptin, and increased intact parathyroid hormone (iPTH) levels [107]. HDF has been associated with a lower incidence of muscle cramps in select patient populations. Both observational and interventional studies, such as those by Karkar et al. and Morena et al., reported a significant reduction in cramp frequency among adult and elderly HDF patients undergoing HVHDF [46,73]. Despite these encouraging findings, the current evidence base is limited, and further well-designed studies are needed to confirm HVHDF’s superiority in cramp prevention.

### 3.4. Improved Biocompatibility, Reduced Inflammation and Oxidative Stress

Each hemodialysis session exposes the patient’s blood to the extracorporeal circuit, initiating mechanical and biochemical responses, including endothelial activation. This cascade begins with vascular access puncture and continues through blood–membrane interaction, which stimulates coagulation and immune pathways—most notably complement activation—contributing to systemic inflammation and oxidative stress [108,109]. Although high-flux HD uses high-permeable membranes similar to HDF, it lacks the convective clearance capacity and fluid purity ensured by online HDF. In contrast, online-HDF systems do not meaningfully trigger complement activation beyond the initial transient contact phase, and they avoid sustained leukocyte or platelet activation. In one comparative study, regardless of membrane type, online HDF did not increase C3a/C5a levels, platelet P-selectin, or activated monocyte counts, indicators that remained consistently low, highlighting the high biocompatibility of the therapy [110].

By comparison, high-flux HD may elicit additional immune stimulation due to the back-filtration of dialysate impurities, including endotoxins and bacterial fragments [111,112]. The detection of anti-endotoxin antibodies in HD patients indicates that these contaminants can cross dialysis membranes, especially high-flux types [112,113,114], triggering complement activation and systemic inflammation. Online HDF mitigates these risks by utilizing ultrapure dialysate, sterile substitution fluid, and high ultrafiltration rates. In addition, convective clearance may also contribute to the removal of circulating complement fragments [38,56] and proinflammatory mediators, enhancing overall biocompatibility. Online HDF also helps prevent transient leukopenia and neutrophil activation that were historically observed with less biocompatible systems. Compared to high-flux dialysis, HVHDF demonstrates superior anti-inflammatory effects due to several factors: the use of ultrapure dialysate and sterile substitution fluid, biocompatible membranes, better hemodynamic stability, improved anemia management, and the more effective clearance of middle- and large-molecular-weight solutes—including cytokines and other inflammatory mediators [60,86,87].

HVHDF has been associated with significant reductions in systemic inflammatory and oxidative stress markers such as high-sensitivity C-reactive protein (hsCRP), interleukin-6 (IL-6), tumor necrosis factor-alpha (TNF-α), soluble CD40 ligand, pentraxin, advanced glycation end products (AGEs), oxidized low-density lipoprotein, and complement activation products [18,26,37,52,60,86,87,114,115,116,117,118]. Additionally, HVHDF downregulates proinflammatory monocyte subsets (CD14+/CD16+) and reduces dendritic cell maturation, effects particularly pronounced in diabetic patients, possibly due to improved autonomic regulation and reduced sympathetic activation [118,119,120]. In a prospective pediatric study, Ağbaş et al. demonstrated that after just three months of HDF therapy, total antioxidant capacity (TAC) increased significantly in children, both with and without residual renal function [86]. Although large-scale head-to-head trials remain limited, the cumulative anti-inflammatory and antioxidative effects of HDF likely contribute to its established cardiovascular and survival benefits, reinforcing its preferential use in patients with elevated inflammatory burden.

### 3.5. Improved Anemia Management

HVHDF offers significant advantages in optimizing anemia management in patients with ESKD, surpassing conventional HD through a multifactorial approach [12] that includes enhanced removal of middle and large molecular weight uremic toxins; reduced systemic inflammation and oxidative stress; improved iron metabolism; more efficient phosphate control; prolonged red blood cell survival. HDF offers a multifaceted approach to improving treatment outcomes and reducing dependence on pharmacological interventions more efficiently than conventional HD [21,121,122,123,124,125,126]. HDF effectively clears larger uremic molecules known to inhibit erythroid progenitor cells. HDF promotes more efficient erythropoiesis by clearing uremic toxins that inhibit erythroid progenitor proliferation [127,128,129], including burst-forming unit–erythroid (BFU-E) suppression factors [130]. Simultaneously, HDF reduces systemic inflammation, lowering circulating interleukin-6 and C-reactive protein levels, decreasing hepcidin synthesis [131,132]. The resulting hepcidin suppression enhances iron mobilization and utilization, reducing functional iron deficiency and the need for both erythropoiesis-stimulating agents (ESAs) and intravenous iron and supporting a more sustained hematological response [133,134]. Clinical evidence, including the REDERT trial and large cohort analyses, confirms that patients on HVHDF demonstrate lower ESA resistance indices and reduced hepcidin levels compared to those on conventional HD [88,89]. This supports a mechanistic link between improved iron metabolism and reduced pharmacologic dependency.

Additional benefits of HDF include more effective phosphate removal, leading to better control of secondary hyperparathyroidism, a known contributor to ESA resistance and bone-marrow suppression. Moreover, HDF has been shown to prolong red blood cell (RBC) survival, thereby stabilizing hemoglobin levels and reducing hemolysis-associated anemia [135,136].

The combined use of HDF with long-acting intravenous ESAs seems to benefit anemia management by reducing the Erythropoietin Resistance Index [8]. However, large-scale RCTs are needed to conclusively determine the magnitude of HDF’s benefit for anemia in ESKD. Overall, HVHDF appears particularly advantageous for patients with inflammation-related ESA resistance, positioning it as a preferred modality for optimizing anemia management in ESKD patients [12].

### 3.6. Preservation of Residual Kidney Function

HVHDF may better preserve residual kidney function compared to conventional HD, primarily through improved hemodynamic stability and a reduction in micro-inflammation [96,137]. The use of ultrapure dialysate and biocompatible membranes has been associated with sustained urine output, with outcomes potentially comparable to those seen in peritoneal dialysis. However, while physiologically plausible, this benefit remains theoretical, as robust confirmation from large-scale RCTs or observational studies is still lacking. Further research is needed to determine the long-term effects of HDF’s on residual kidney function.

### 3.7. Reduction in Skin Hyperpigmentation

Online HDF has been associated with a significant decrease in skin hyperpigmentation among patients with ESKD, likely due to its superior clearance of β2-microglobulin and other pigmentary middle-molecular-weight toxins (e.g., melanin). Lin et al. reported improved skin pigmentation in ESKD patients with increased and more frequent HDF, though without objective colorimetric assessment [138]. Moon et al. subsequently demonstrated that HDF significantly reduced skin pigmentation compared to low-flux HD, identifying HDF as an independent predictor of decreased melanin index in the forehead region [139]. Shibata et al. confirmed that HD patients exhibited darker skin than healthy controls, and those on online HDF experienced notable skin lightening, coinciding with a reduction in β2M levels [140]. These findings suggest that MMW accumulation contributes to uremic skin changes and that HDF offers a dermatological benefit beyond traditional uremia management.

## 4. Mid-Term Intermediate Outcomes

Over months to years, HDF demonstrates sustained clinical benefits, improving patient well-being and reducing dialysis-related complications.

### 4.1. β2-Microglobulin Amyloidosis and Joint Symptoms Control

HDF offers superior removal and lower circulating levels of β2M compared to conventional hemodialysis, especially when high convection volumes are delivered [39,40,41,42,43,44]. This enhanced clearance is associated with a reduced risk of dialysis-related amyloidosis (DRA), manifesting as carpal tunnel syndrome, bone cysts, and joint pain [11,141]. Since β2M clearance increases with convection volume [40,142], HVHDF remains the most effective modality for its removal. Importantly, DRA also contributes to systemic complications, including cardiovascular disease and autonomic dysfunction [42,143,144]. The combined use of ultrapure dialysate, biocompatible membranes, and effective convective clearance facilitates these improvements [11,145]. HVHDF is particularly indicated in patients with β2M levels above 27 mg/L or symptomatic DRA [10]. Clinical improvement in joint mobility and pain relief is frequently reported after switching from HD to HVHDF [10,146], supporting its utility of HVHDF in advanced amyloidosis and improving quality of life.

### 4.2. Improved Nutritional Status

HVHDF is consistently associated with better nutritional outcomes in patients with ESKD. Observational studies show increased appetite, higher protein intake, and preservation of lean body mass [11,90,147]. Compared to high-flux HD, patients on HVHDF show improved body cell mass and nutritional markers [90,91]. A key mechanism is the superior clearance of proinflammatory and anorexigenic middle molecules, particularly leptin, which is often elevated in dialysis patients and contributes to appetite suppression and inflammation [11,48,148] This enhanced removal helps mitigate the Malnutrition-Inflammation Complex Syndrome (MICS), a condition characterized by protein-energy wasting, chronic inflammation, and elevated levels of cytokines such as IL-6, TNF-α, and IL-1β. By interrupting this cycle, HVHDF supports better maintenance of serum albumin and prealbumin levels, reduces muscle catabolism with improved preservation of lean tissue mass, and improves functional capacity, especially in patients with inflammatory cachexia [90,149,150,151]. Although HVHDF may induce mild losses of amino acids, water-soluble vitamins, and micronutrients, these can typically be corrected with oral supplementation [11,152,153].

### 4.3. Reduced Infection Risk

Infectious complications are a leading cause of morbidity and the second most common cause of death in ESKD patients after cardiovascular disease [154,155]. This elevated risk stems from uremia-induced immune dysfunction, characterized by impaired innate and adaptive immunity due to the accumulation of middle and large-molecular-weight uremic toxins [150,151,156,157,158]. Additional factors include advanced age, diabetes, central venous catheter use, hypoalbuminemia, comorbidities, inadequate hygiene practices, and latent disease [159,160,161,162,163,164]. HVHDF offers a promising strategy to reduce infection risk by enhancing the clearance of immune-impairing uremic toxins [56]. Its superior hemodynamic stability may also reduce episodes of intestinal ischemia and bacterial translocation, an underrecognized pathway for systemic infection in dialysis patients [7,165,166]. HVHDF is associated with stronger vaccine-induced immune responses than conventional HD. Studies show more sustained seroprotection and higher lymphocyte proliferation in response to influenza A vaccination [10,167] and higher, more durable antibody titers after SARS-CoV-2 vaccination [10,168,169,170,171]. Robust clinical data support these immunological benefits.

The ESHOL study demonstrated a 55% reduction in infection-related mortality and a 22% reduction in infection-related hospitalizations in the HVHDF group compared to high-flux HD [10,34]. Similarly, the CONVINCE trial demonstrated a lower hazard ratio for infection-related death in patients treated with HVHDF (HR 0.69; 95% CI, 0.49–0.96), including COVID-19 related deaths [2]. A meta-analysis by Vernooij et al., pooling data from five randomized controlled trials (*n* = 4153 patients), reported that patients receiving convection volumes ≥ 23 L/session had a 49% lower risk of infection-related mortality compared to HD (adjusted HR 0.51; 95% CI, 0.28–0.93) [3].

### 4.4. Cardiovascular Benefits

Growing evidence supports the role of HVHDF in reducing all-cause mortality and cardiovascular mortality in ESKD patients [8]. The pathogenesis of cardiovascular disease in this population is driven by chronic inflammation, oxidative stress, and the retention of uremic toxins, which accelerates vascular aging, promotes arterial stiffness, impairs endothelial function, and contributes to vascular calcification [8]. HVHDF mitigates these mechanisms through both direct and indirect effects: Direct cardiovascular effects include reduced intradialytic hypotension, enhanced hemodynamic stability (independent of sodium balance), improved cardiac remodeling, and attenuated left ventricular hypertrophy [60,70,71,72,79,81,172]. HVHDF also improves endothelial function, reduces arterial stiffness, slows the progression of atherosclerosis, and lowers systemic inflammation and oxidative stress [86,87,173,174,175,176]. Moreover, HVHDF may lower sympathetic overactivity and reduce arrhythmogenic risk [177,178]. The PBUTs indoxyl sulfate and p-cresyl sulfate are strongly associated with endothelial dysfunction, inflammation, vascular calcification, and increased cardiovascular and all-cause mortality. It has been demonstrated that HVHDF enhances the clearance of indoxyl sulfate and p-cresyl sulfate [63,64,65]. Post hoc analysis of the HDFit trial over six months showed a monthly reduction in indoxyl sulfate in pre-dialysis plasma and a significant decrease in p-cresyl sulfate among patients with HVHDF (convection volume > 27.5 L) compared to high-flux HD [65]. This enhanced middle-molecule clearance leads to reductions in systemic inflammation and improved endothelial health, which are central to HDF’s cardiovascular benefits [65]. Indirect effects contributing to cardiovascular health include better anemia control [88,89,90], improved nutritional status [90,91], increased physical activity [92], enhanced quality of life [91,93,94,95], and preservation of residual kidney function [96].

### 4.5. Peripheral Neuropathy Improvements

Peripheral neuropathy is the most common long-term neurological complication in ESKD, typically presenting as symmetrical, distal sensorimotor neuropathy predominantly affecting the lower limbs. Symptoms include paresthesia, sensory loss, reduced reflexes, muscle weakness, insomnia, irritability, pruritus, and restless legs syndrome (RLS) [179,180,181,182]. The pathogenesis involves the accumulation of neurotoxic middle molecules, such as indoxyl sulfate, p-cresyl sulfate, β2-microglobulin, PTH, and [183,184,185] oxidative stress [185], leading to demyelination and axonal degeneration. Risk factors include prolonged dialysis duration, suboptimal dialysis dose, diabetes, micronutrient deficiencies, and advanced age.

Preliminary evidence suggests that HVHDF may slow the neuropathy progression by enhancing the clearance of neurotoxic middle MW toxins [186]. Patients transitioning from HD to HDF have reported improvements in pruritus [93,187,188] and RLS symptoms. In a report by Sakurai et al., two patients with refractory RLS experienced marked improvement with HVHDF, which correlated with achieving an α1-microglobulin (α1-MG) removal rate ≥ 40% [59]. Notably, symptoms recurred upon switching back to conventional HD or when the α1-MG clearance declined [59], suggesting that α1-MG may serve as a therapeutic target marker in RLS management and that HVHDF may offer a promising non-pharmacologic therapy in these cases. However, the FINESSE RCT conducted in Australia did not confirm these findings, which reported no significant differences in neuropathy progression between HDF and HD [189]. Nonetheless, methodological limitations (nerve conduction measurements) and potential biases may have influenced the outcomes.

While symptomatic relief is achievable with HVHDF, particularly for pruritus and RLS, structural nerve damage from longstanding uremia remains largely irreversible [189], underscoring the importance of early intervention and the start of HVHDF.

### 4.6. Cognitive and Quality-of-Life Benefits

Cognitive impairment and reduced health-related quality of life (HRQoL) are highly prevalent yet frequently underrecognized complications in the dialysis population [190]. Cognitive defects span memory, executive function, attention, visuospatial skills, emotional well-being, social engagement, and physical performance [191,192,193]. Prevalence estimates vary, but moderate to severe impairments affect up to 70% of patients over 55 years old receiving HD, and significant deficits can occur at any age [191,192,193]. The etiology is multifactorial, involving traditional risk factors (e.g., aging, depression, vascular disease), ESKD-related contributors (e.g., chronic inflammation, oxidative stress, mineral bone disorders, anemia), and dialysis-specific mechanisms such as recurrent intradialytic hypotension, cerebral hypoperfusion, and the accumulation of neurotoxic solutes [194,195,196,197,198,199,200,201,202,203,204,205]. These synergistic insults lead to cerebral ischemia, white matter damage, and progressive neurocognitive decline. HVHDF may mitigate these effects by improving intradialytic hemodynamic stability, enhancing the clearance of neurotoxic middle-molecular-weight molecules, and reducing systemic inflammation and oxidative stress.

The CONVINCE trial demonstrated a significantly slower rate of cognitive decline in patients treated with HVHDF compared to those receiving high-flux HD [4], with the greatest preservation seen in cognitive function scores. These findings suggest a potential neuroprotective role of HVHDF. Beyond cognition, HVHDF has demonstrated positive impacts on broader quality-of-life measures. The HDFit trial reported increased physical activity, reflected by higher daily step counts, among patients on HDF, although no change was noted in sleep duration [92,206].

In CONVINCE, HRQoL was assessed every 3 months over an extended period using the PROMIS^®^ tool across eight domains, including physical and cognitive function, fatigue, pain interference, and social participation [2,4]. While cognitive function declined in both treatment arms over time, the decline was significantly attenuated in the HVHDF group. In addition to cognitive outcomes, the study provided robust data to show that treatment with HVHDF led to better physical function, social interaction, and pain interference than standard high-flux HD [4]. These findings are further supported by the EuDial Working Group, which recognizes HVHDF as potentially superior to high-flux HD in preserving patient-reported physical and cognitive dimensions of health-related quality of life (HRQoL). Specifically, HVHDF has been associated with better maintenance of cognitive function, reduced symptom burden, and improved physical activity levels when compared to high-flux HD [7]. By enhancing intradialytic hemodynamic stability, reducing systemic inflammation, uremic toxin load, improving nutritional status, and better preserving the lean tissue mass, HVHDF appears to exert a protective effect on both neurological function and physical capacity—two critical components of patient well-being often compromised in individuals with end-stage kidney disease.

Karkar et al. assessed patient-reported outcomes using the KDQOL-SF questionnaire and found that patients receiving high-efficiency post-dilution HDF for 24 months reported significantly lower levels of post-dialysis fatigue compared to those on high-flux HD [93]. In addition to reduced fatigue, patients treated with HDF experienced improvements in overall mood, body energy, and physical function, including better perceptions of physical fitness and walking ability. HDF was also associated with better control of cramps, joint pain, and stiffness, suggesting a broader impact on musculoskeletal symptoms and well-being that may contribute to increased physical activity and quality of life in dialysis patients [93]. A significant reduction in post-dialysis fatigue and improvement in physical activity were observed in the short and long term for HDF pediatric patients [7,176,207].

Uremic pruritus (UP) is a common and distressing symptom experienced by patients with ESKD, as documented in multiple studies [187,208,209]. A cross-sectional global survey has shown that 42% of patients with ESKD experience pruritus of varying severity, a symptom that significantly worsens HRQoL [210,211]. UP is also strongly associated with reduced sleep quality, depressive symptoms, and increased mortality in this population [210]. Multiple pathophysiological mechanisms have been proposed to explain UP. These include hypervitaminosis A, dry skin, secondary hyperparathyroidism, iron deficiency, and disturbances in mineral balance, particularly involving phosphate, magnesium, and calcium [212]. Microinflammation, elevated histamine levels, and histamine binding to H4 receptors have been implicated in this condition. UP is increasingly recognized as a multifactorial condition, involving complex interactions between opioid receptor dysregulation, mast cell activation, increased skin surface pH, and biocompatibility issues related to dialyzer membrane components [213,214]. HVHDF has shown potential to improve UP symptoms [93] through its combined anti-inflammatory, detoxifying, and biocompatible effects; however, the evidence remains limited and indirect.

Taken together, these data highlight that HVHDF improves survival and physiological parameters and helps preserve cognitive function, physical independence, and overall well-being in patients with ESKD.

## 5. Long-Term Outcomes

**Figure 1:**
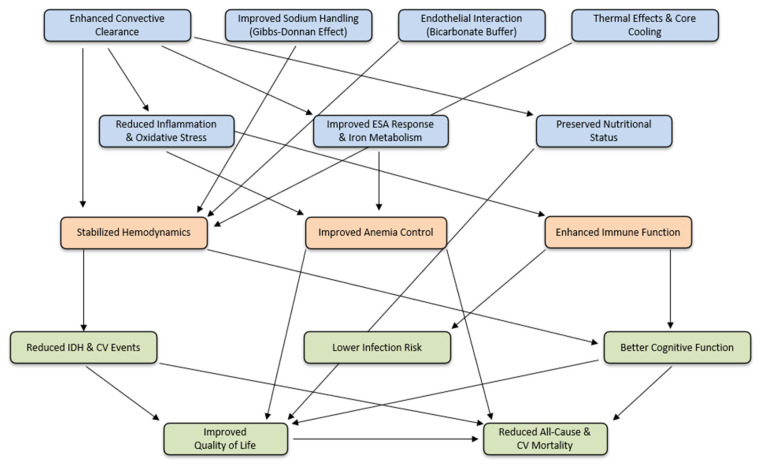
Mechanistic cascade of high-volume HDF: from enhanced solute clearance to survival benefit in ESKD patients CV: cardiovascular; IDH: intradialytic hypotension; ESA: erythropoietin-stimulating agents.

While the short- and medium-term benefits of HDF are important from the patient perspective, the cumulative physiological advantages of HVHDF translate into superior long-term outcomes compared to high-flow HD (Figure 1).

### 5.1. Randomized Controlled Trials

Numerous RCTs, meta-analyses, and RWE studies have evaluated the effect of HDF/HVHDF on all-cause mortality versus conventional HD. While RCTs remain the gold standard for establishing causal efficacy, their generalizability is often limited by strict inclusion criteria, protocol standardization, and intensive monitoring, conditions that do not reflect the complexity of routine ESKD care [215,216,217,218,219,220,221]. In contrast, RWE, derived from observational cohorts, registries, and clinical databases, provides valuable complementary insights. As emphasized by Canaud et al., RWE is essential for assessing treatment effectiveness, scalability, and safety in real-world practice [11]. This is particularly relevant in ESKD, where patient heterogeneity, comorbidities, and care variability complicate the application for RCT findings to daily nephrology [11,12]. Integrating both RCT and RWE is thus essential for a comprehensive assessment of HDF’s clinical value.

Recent evidence suggests that HDF, when delivered with high convection volumes (>23 L/session), improves survival compared to high-flux HD. In 2025, the EuDial published a consensus statement synthesizing findings from systematic reviews and expert evaluations of both adult and pediatric populations [7]. The panels issued 22 consensus statements addressing survival, cardiovascular events, health-related quality of life, and biochemical markers. A key conclusion statement is that HVHDF is associated with reduced all-cause and cardiovascular mortality, particularly in patients with favorable clinical profiles and reliable vascular access. The benefit is most consistently observed in patients dialyzed via arteriovenous fistula, underscoring the importance of both patient selection and optimized convection dosing [7].

The mortality benefit of post-dilution HDF has been evaluated in six major European RCTs, Italian Convective Study, CONTRAST, Turkish, ESHOL, FRENCHIE, and CONVINCE, with outcomes largely influenced by the level of convection volume achieved [2,33,34,70,73,222] (Table 3).

While the CONTRAST and Turkish trials did not demonstrate an overall survival advantage of HDF compared to conventional HD, both reported significant mortality reductions in subgroup analyses achieving higher convection volume. In CONTRAST, patients receiving > 21.9 L/session had a 38% lower risk of death (HR = 0.62; 95% CI: 0.41–0.83) [223]. Similarly, the Turkish trial reported a 29% mortality reduction for those with substitution volumes > 17.4 L/session (HR = 0.71; 95% CI: 0.07–0.71; *p* = 0.01) [33]. The ESHOL trial (2013), a multicenter, open-label RCT, provided robust evidence supporting the survival benefit of post-dilution HVHDF. Among 906 prevalent ESKD patients, HDF reduced all-cause mortality by 30% (HR = 0.70; 95% CI: 0.53–0.92; *p* = 0.01) and cardiovascular mortality by 33% (HR = 0.67; 95% CI: 0.44–1.02; *p* = 0.06) [34]. Post hoc analyses revealed a dose–response relationship: patients achieving 23–25 L/session had a 40% lower mortality (HR = 0.60; 95% CI: 0.39–0.90), while those exceeding 25 L/session saw a 45% reduction (HR = 0.55; 95% CI: 0.34–0.84) [34]. By contrast, the FRENCHIE trial (2017) found no significant difference in mortality between HD and post-dilution HDF groups, likely due to lower achieved convection volumes compared to ESHOL [73]. The CONVINCE trial (2023), a large, multicenter RCT funded by the European Union’s Horizon 2020 program, enrolled 1360 ESKD patients from 61 dialysis centers across eight European countries (Spain, Romania, Germany, Portugal, France, Hungary, the Netherlands, and the UK) [2]. Patients randomized to post-dilution HVHDF (≥23 L/session) experienced a 23% reduction in all-cause mortality (HR = 0.77; 95% CI: 0.65–0.93) after a median follow-up of 30 months, while also achieving higher dialysis dose (spKt/V) compared to the HD group [2]. Notably, HVHDF was associated with significant improvements in health-related quality of life [4].

The ongoing “High-Volume Hemodiafiltration vs. High-Flux Hemodialysis Registry Trial (H4RTin the UK) is designed further to assess the clinical benefits of HVHDF versus HD [223]. This registry-based RCT targets convection volumes > 23 L per session in post-dilution mode [223], with results expected by 2026.

### 5.2. Meta-Analyses: Expanding the Case for HVHDF

Before 2016, several meta-analyses assessed convective dialysis techniques, but lacked a specific on HDF [11]. Instead, they broadly compared convection-based therapies including hemofiltration (diafiltration), acetate-free biofiltration (AFB), and paired filtration dialysis (PFD), and provided limited insights into the impact of convective volume on outcomes [74,173,178,224].

European Pooling Project. The first major meta-analysis targeting HDF was in 2016 the European Pooling Project, an individual patient data (IPD) meta-analysis, combining data from four randomized controlled trials (CONTRAST, Turkish, ESHOL, and FRENCHIE; N = 2793) comparing post-dilution HDF (*n* = 1400) with conventional HD (*n* = 1393) to assess long-term clinical outcomes [6]. Over a median follow-up of 2.5 years, patients receiving > 23 L per 1.73 m^2^ body surface area per session experienced a 22% reduction in all-cause mortality (HR = 0.78; 95% CI: 0.62–0.98) and a 31% reduction in cardiovascular mortality (HR = 0.69; 95% CI: 0.47–1.00), after adjustment for key covariates [6]. Comprehensive IPD meta-analysis by Vernooij et al. In 2024, Vernooij et al. conducted an updated a pooled IPD analysis of five European RCTs (CONTRAST, Turkish, ESHOL, FRENCHIE, and CONVINCE; N = 4153; 2083 HDF; 2070 HD) [3]. HVHDF was associated with a 16% reduction in all-cause mortality (HR = 0.84 (95% CI: 0.74–0.95) and a 22% reduction in cardiovascular mortality (HR = 0.78; 95% CI: 0.64–0.96) [3]. Subgroup analyses suggested enhanced survival in patients aged ≥ 65 years, those without diabetes or cardiovascular disease, and individuals with dialysis vintage ≥ 30 months. Conversely, no significant mortality benefit was observed in younger patients (<65 years), those with diabetes, or patients with biochemical markers of malnutrition (e.g., serum albumin < 4.0 g/dL). Although no significant interaction was found, a clear dose–response relationship emerged: greater convection volume was associated with lower mortality risk [3].

### 5.3. Reinforcement from Real World Evidence and Dose–Response Across Observational Studies

Several RWE studies corroborate the survival benefit of HDF seen in RCTs and meta-analyses, consistently showing a dose–response association between substitution/convective volume and relative survival rate [15,20,225,226,227,228,229,230,231]. Benefits are most pronounced when substitution/convective volumes exceed 21/23 L per session, respectively, and have exhibited the most favorable effect on mortality outcomes [225,226,227,228,229,232].

In 2006, the DOPPS study first reported improved survival with substitution volumes greater than 15 L [20]. The French National Registry showed reduced all-cause (HR = 0.84) and cardiovascular mortality (HR = 0.73) in HDF patients [230]. The Australia and New Zealand Dialysis and Transplant Registry (ANZDATA) reported similar findings; HR for all-cause was 0.79 in Australia and 0.88 in New Zealand, with cardiovascular mortality significantly lower in the Australian HDF cohort (HR 0.78) [231]. The Japanese Society for Dialysis Therapy Renal Data Registry database supports these results, indicating that pre-dilution HDF was associated with a reduction in all-cause mortality (HR 0.83), particularly when high-volume convective doses were employed [15]. In Latin America, propensity score-matched cohort studies from Brazil and Colombia demonstrated substantial mortality reductions (HR = 0.71 and 0.45, respectively) among HDF-treated patients [233,234].

Recently, two observational studies evaluated the real-world effectiveness of HDF and HVHDF compared to high-flux HD in a large, unselected patient population treated at Fresenius Medical Care (FME) NephroCare clinics across Europe, Asia, Africa, and Latin America [235,236]. The analyses were based on data extracted from EuCliD (European Clinical Dialysis Database), a standardized electronic medical record system used in FME NephroCare clinics outside the United States [237,238]. In the first study, Zhang et al. assessed the real-world effectiveness of HVHDF compared to high-flux HD on 85,117 adult patients treated in FMC NephroCare clinics across 23 countries between 2019 and 2022 (Bosnia and Herzegovina, Croatia, Czech Republic, Estonia, Finland, France, Hungary, Italy, Kazakhstan, Kyrgyzstan, Netherlands, Poland, Portugal, Romania, Russia, Serbia, Slovakia, Slovenia, South Africa, Spain, Sweden, Turkey, and Ukraine) [235]. The analysis confirmed a 22% reduction in all-cause mortality with HDF versus HD; a 30% mortality reduction for those receiving HVHDF (≥23 L convection volume per session); a 31% lower risk of cardiovascular mortality for HDF [235]. Consistent benefits across subgroups regardless of age, dialysis vintage, diabetes, or cardiovascular disease status [235]. Sustained survival advantage during the COVID-19 pandemic, regardless of infection status [235]. Robustness of findings confirmed via sensitivity analyses, adjusting for demographics, clinical parameters, and country-level variation [235]. In the second study Strogoff-de-Matos et al. assessed mortality risk among Brazilian patients with kidney failure, comparing outcomes between those treated with HDF and those receiving high-flux HD [236]. The cohort included 8391 end-stage kidney disease (ESKD) patients treated at 29 dialysis facilities in Brazil between 1 January 2022, and 31 December 2023: 6787 received only high-flux HD, while 2836 received HDF. In a time-updated analysis, patients treated with HDF had a significantly lower adjusted risk of all-cause mortality compared to those on HD (HR 0.73) [236]. HDF was also associated with reduced cardiovascular mortality but not with lower infection-related mortality. The mortality benefit was consistent across subgroups and more pronounced in patients under 65 years (HR 0.56) compared to those aged 65 or older (HR 0.82) [236].

The growing body of evidence from RCTs, IPD meta-analyses, and real-world studies consistently supports the mortality benefit of post-dilution HVHDF, particularly when adequate convection volumes are delivered. A grade, dose–response relationship between convection volume and survival underscores the clinical importance of achieving high convective efficiency (>23 L/session) in routine practice [3].

## 6. Economic and Implementation Considerations

The CONVINCE trial not only demonstrated the clinical superiority of HVHDF compared to high-flux HD, but also evaluated its cost-effectiveness across eight European countries [239]. Using a Markov model, the cost–utility analysis estimated incremental cost-effectiveness ratios (ICERs) ranging from EUR 31,898 to EUR 37,344 per quality-adjusted life year (QALY) over a two-year horizon, and from EUR 27,068 to EUR 36,751 per QALY over a lifetime horizon.

When additional life-year costs were excluded, the ICER dropped to EUR 13,231 per QALY, with a >90% probability of cost-effectiveness at a EUR 50,000 threshold [239]. When intervention costs related to extended survival were excluded, the ICER dropped significantly to EUR 13,231 per QALY, underscoring HDF’s favorable economic profile [239]. Key cost drivers included dialyzer disposables, higher water and energy use, and extended dialysis sessions. However, these were partially offset by reduced medication needs and improved quality of life. Hospitalization costs differed modestly, and further analysis is needed to understand the impact on recurrent admissions. Cost-effectiveness varied by country, emphasizing the need for local economic adaptation.

The CONVINCE trial provides compelling evidence that HVHDF is a cost-effective therapeutic strategy for ESKD. Despite higher upfront resource utilization, the substantial survival benefit and favorable cost-per-QALY estimates support HDF as a clinically and economically sound investment in renal replacement therapy.

Canaud et al., using real-world data and simulation modeling, demonstrated that optimally prescribed post-dilution HVHDF is more environmentally sustainable than high-flux HD [25]. By lowering the dialysate flow rate (e.g., 430 mL/min with a Qd:Qb ratio of 1.1) and utilizing automated ultrafiltration and substitution controls, HVHDF maintains superior clearance of both small and middle molecules while reducing water and dialysate consumption [25]. These findings support HVHDF as a more resource-efficient and clinically effective dialysis modality.

While the clinical benefits of HVHDF are well-supported by randomized trials and real-world data, it is important to acknowledge certain practical limitations. HVHDF implementation requires specialized machines, ultrapure water systems, and optimized vascular access, resources not universally available. Achieving high convection volumes depends on blood flow rates, patient characteristics, and machine settings, which can potentially introduce variability in practice.

Emerging alternatives such as expanded hemodialysis using medium cut-off (MCO) dialyzers or super high-flux membranes offer equivalent middle-molecule clearance without requiring online sterile substitution fluid. Early studies show a promising clearance profile [240]. However, intermediate and hard outcome data (e.g., intradialytic hemodynamic stability, anemia management, enhanced immune function, all-cause mortality, cardiovascular events, infection-related mortality) from large randomized controlled trials and real-world longitudinal studies are not yet available for these modalities. While they represent promising approaches, their clinical efficacy remains to be validated against the robust survival and quality-of-life benefits already demonstrated with HVHDF. According to Blankestijn, current clinical studies provide limited justification for transitioning from high-flux to MCO dialyzers [241]. Furthermore, there is insufficient understanding of how prescription parameters can be optimized to dose this therapy [241]. The indications for use also remain undefined and uncertain [241].

## 7. Conclusions

Post-dilution HVHDF represents a significant advancement in renal replacement therapy, consistently demonstrating superior outcomes compared to high-flux HD. When delivered with adequate convection volumes (≥23 L/session), HVHDF is associated with significant reductions in all-cause and cardiovascular mortality, as confirmed by recent randomized controlled trials, individual patient-level meta-analyses, and large-scale real-world evidence. Importantly, the clinical benefits of HVHDF extend beyond survival. HVHDF offers broad clinical advantages, including improved hemodynamic stability, enhanced toxin removal, inflammation control, and better anemia management, all of which contribute to a superior quality of life. These effects are dose-dependent and consistent across heterogeneous patient populations, underscoring the importance of optimized delivery and automated system implementation. Given the robustness and consistency of evidence, HVHDF should be considered not merely as an alternative to high-flux HD but as a preferred standard of care. Wider implementation, especially in regions where uptake has been limited or has not yet started, will require targeted education, infrastructure, and technological support, as well as integration into outcome-driven clinical frameworks. Future efforts should focus on embedding HVHDF into personalized, value-based care models, emphasizing survival, symptom control, and patient-reported outcomes. Policymakers, payers, and advocacy groups must acknowledge HVHDF’s clinical and economic value and collaborate to ensure equitable access, appropriate reimbursement models, and sustainable implementation at scale.

## 8. Future Perspectives

As HVHDF continues to gain clinical acceptance and technological feasibility worldwide, several important challenges and future directions must be addressed to optimize its implementation and sustainability.

Personalized prescription: While current evidence supports the broad use of post-dilution HVHDF across most maintenance dialysis populations, the primary limitation relates to blood flow rate and blood viscosity. Further studies are needed to refine patient stratification criteria. Identifying individuals most likely to benefit—such as those with a high inflammatory burden, cardiovascular risk, or poor toxin clearance—will help tailor therapy and maximize clinical value. Conversely, frail or hemodynamically unstable patients may require modified protocols or staged transitions.

Education, training, and clinical adoption: The successful integration of HVHDF into routine clinical care depends on the development of comprehensive training programs for nephrologists, nurses, and dialysis technicians. Targeted workshops, reference centers, and structured clinical training can bridge knowledge gaps and accelerate adoption. Standardizing protocols (e.g., treatment time ≥ 240 min, convective volume ≥ 23 L) and utilizing integrated systems with automated infusion control can improve operational efficiency and help mitigate the learning curve.

Balancing upfront investment with long-term gains: Despite the higher initial investment in infrastructure and equipment, HVHDF offers substantial long-term benefits, including reduced hospitalizations, improved survival, lower medication requirements, and enhanced quality of life—supporting its overall financial sustainability.

Integration with Patient-Reported Outcomes and Value-Based Care: Future research should emphasize the inclusion of validated patient-reported outcome measures (PROMs), such as fatigue, pruritus, sleep quality, and physical functioning. These data will support the alignment of HVHDF with patient-centered care principles and value-based performance frameworks.

Stakeholder collaboration and innovation networks: Cross-sector collaboration among clinicians, researchers, industry stakeholders, policymakers, and nephrology societies is critical to advancing innovation, defining clinical standards, and supporting global dissemination. Multinational initiatives such as the CONVINCE study promote shared learning, evidence generation, and best-practice implementation across diverse care settings.

Patient engagement and shared decision-making: Involving patients and patient advocacy groups in the selection of dialysis modalities ensures that treatment decisions reflect patients’ values and preferences. Accessible educational materials outlining the potential benefits and limitations of HVHDF can foster informed decision-making, increase patient satisfaction, and promote engagement in therapy.

## Figures and Tables

**Table 1:** HDF and high-volume HDF effects on short, mid-, and long-term clinical outcomes.

Time Frame	Outcome Domains	HDF Key Findings
**Short Term**	↑ toxin clearance	↑ removal of small/MM.
within days	↑ hemodynamic stability	↓ IDH, ↑ thermal balance, ↓ inflammation,…
to weeks	↓ inflammation	↓ hsCRP, ↓ IL-6, ↓ TNF-α, ↓ β2M, ↓ pentraxin…
	↓ oxidative stress	↓ AGEs, ↓ oxidized LDL, ↑ TAC, …
	↑ anemia management	↓ ESA resistance/hepcidin, ↑ RC survival
	↑ kidney protection	↑ hemodynamic stability, ↓ inflammation
	↓ intradialytic cramps	↓ IDH (?)
	↓ skin hyperpigmentation	↓ melanin
**Mid Term**	↓ amyloidosis, ↓ JP	↓ β2M, ↓ inflammation
months to 2	↑ nutritional status	↑ LT mass, ↑ physical activity, ↓ leptin
years	↓ infection risk	↓ uremic toxins, ↓ intestinal ischemia, …
	↑ CV benefits	Direct and indirect effects on CV system
	↑ peripheral neuropathy	↓ oxidative stress, ↓ indoxyl sulfate, ↓ β2M, …
	↓ cognitive impairment	↓ neurotoxic MM, ↓ cerebral ischemia, …
	↑ HRQoL improvement	↑ physical function, ↑ social participation, …
**Long Term**	↓ all-cause mortality	Convection volume ≥ 23 L/session
>2 years	↓ CV mortality	Convection volume ≥ 23 L/session
	Cost-effectiveness	↓ cost–QALY in economic modeling

↑: improved/increased; ↓: reduced/decreased; MM: middle molecules; IDH: intradialytic hypotension; hsCRP: high-sensitive C-reactive protein; IL-6: interleuchin-6; TNF-α: tumor necrosis factor-*α*; β2M: Beta 2 microglobulin; AGEs: advanced glycation end products; oxidized LDL: oxidized low-density lipoprotein; TAC: total antioxidant capacity; ESA: erythropoietin stimulating agents; RC: red cells; JP: joint pain; LT: lean tissue; CV: Cardiovascular; QALY: quality-adjusted life years.

**Table 2:** Main middle-molecular-weight substances (MMWs) and middle protein-bound uremic toxins (PBUTs) removed by HDF.

MMWs and PBTUs	MW (Da)	Clinical Relevance
Insulin [35]	5800	Glucose metabolism
PTH Fragments [32,36]	9000	CKD-MBD
AGE Products [26,37]	>10,000	Oxidative vascular damage
Complement C3a/C5a [38]	11,500	Inflammation, immune response
Beta 2-Microglobulin [39,40,41,42,43,44,45,46]	11,800	Amyloidosis, inflammation
Leptin [47,48]	16,000	Appetite regulation
TNF-α [49]	17,000	Systemic inflammation
Myoglobin [50,51]	17,000	Rhabdomyolysis marker
Interleukin-1 [52]	17,000	Inflammation, immune signaling
Retinol-Binding Protein [53]	21,000	Insulin resistance
FLC K/L [50,54,55]	22,000	Inflammation, dyscrasias
Beta-trace Protein [50]	23,000	GFR biomarker
Complement Factor D [56]	24,000	Complement activation
Hepcidin [57,58]	25,000	Iron regulation
α-1 Microglobulin [59]	26,000	Tubular injury, oxidative stress
Interleukin-6 [60]	26,000	Inflammation, CV risk
FGF 23 [61,62]	32,000	CKD-MBD, vascular calcification
α-1-Acid Glycoprotein [50]	43,000	Acute-phase protein
PB p-Cresyl Sulfate [63,64,65]	188	Inflammation, atherosclerosis
PB Indoxyl Sulfate [63,64,65]	213	Vascular calcification, ox. stress

PTH: Parathyroid hormone; AGE: advanced glycation end; TNF-α: tumor necrosis factor-*α*; FLC K/L: Free Light Chains Kappa/Lambda; FGF: Fibroblast Growth Factor; PB: protein-bound; ox.: oxidative.

**Table 3:** Six randomized controlled trials have been published in recent years.

Study	Country	Sample Size (HD/HDF)	Sub/Conv Volume (L/session)	Primary Outcome	Key Findings
ICS [70]	Italy	70/40	Sub: 30–40 (pre)	ISH	↓ ISH 50.9% with HDF
CONTRAST [223]	NL-CA	356/358	Sub: 19.8	All-cause mortality	No difference overall, but benefit with high-volume HDF
Turkish [33]	Turkey	391/391	17.2/19.5	All-cause mortality + CV event	No difference overall, better survival in high-efficiency HDF
ESHOL [34]	Spain	450/456	21.8/23.9	All-cause mortality	30% lower all-cause mortality in HDF
FRENCHIE [73]	France	191/190	20/21	Intradialytic tolerance	Better tolerance; no difference in mortality
CONVINCE [2]	M	677/683	23.0/25.5	All-cause mortality	HVHDF ↓ all-cause morta-lity by 23% (HR 0.77)

ICS: Italian Convective Study; NL: the Netherlands; CA: Canada; Sub/Conv Volume: substitution/convection volume. ISH: intradialytic symptomatic hypotension. ↓: reduced; M: multinational (Portugal, Spain, France, United Kingdom, Germany, the Netherlands, Hungary, Romania).

## Data Availability

No new data were created or analyzed in this study.
